# Supplementary material for: Tailored Multiplex Real-Time RT-PCR with Species-Specific Internal Positive Controls for Detecting SARS-CoV-2 in Canine and Feline Clinical Samples
Source: Animals (Basel). 2023 Feb 9;13(4):602. doi: 10.3390/ani13040602 (PMC9951688; doi:10.3390/ani13040602)
Supplement: Supplementary file 1 [file animals-13-00602-s001.zip › Supplementary Figure S1.pdf]

Supplementary Figure S1

(A)

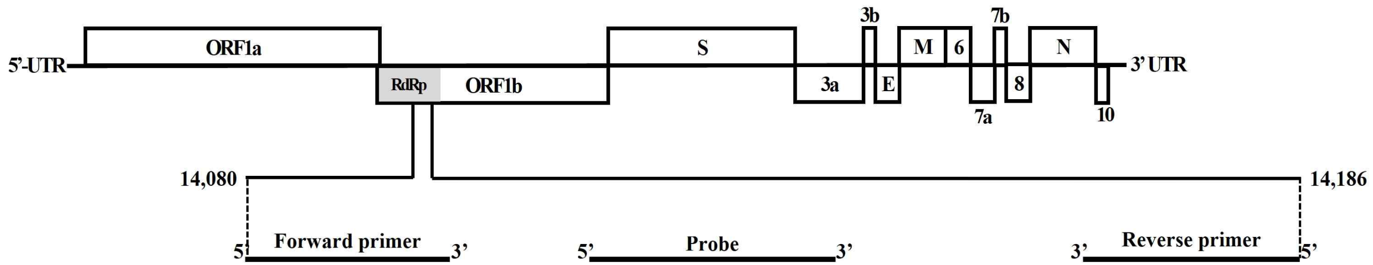

Group	Sequence	Alignment
	mRT-qPCR	----GGTAACTGGTATGATTTCGGT--------------CACGCCAGGTAGTGGAGTTCCTGTT-------------------------TGCCTATATTAACCTTGACCAG----
Wuhan-Hu-1 →	SARS-CoV-2|-|NC_045512|	CAAT.....................GATTTCATACAAAC.........................GTAGATTCTTATTATTCATTGTTAA......................GGCT
Canine SARS-CoV-2	SARS-CoV-2|Alpha|EPI_ISL_5194358|	CAAT.....................GATTTCATACAAAC.........................GTAGATTCTTATTATTCATTGTTAA......................GGCT
	SARS-CoV-2|Delta|EPI_ISL_8900015|	CAAT.....................GATTTCATACAAAC.........................GTAGATTCTTATTATTCATTGTTAA......................GGCT
	SARS-CoV-2|Delta|EPI_ISL_7845316|	CAAT.....................GATTTCATACAAAC.........................GTAGATTCTTATTATTCATTGTTAA......................GGCT
	SARS-CoV-2|Delta|EPI_ISL_4296308|	CAAT.....................GATTTCATACAAAC.........................GTAGATTCTTATTATTCATTGTTAA......................GGCT
	SARS-CoV-2|Delta|EPI_ISL_9906009|	CAAT.....................GATTTCATACAAAC.........................GTAGATTCTTATTATTCATTGTTAA......................GGCT
	SARS-CoV-2|Omicron|EPI_ISL_13101428|	CAAT.....................GATTTCATACAAAC.........................GTAGATTCTTATTATTCATTGTTAA......................GGCT
	SARS-CoV-2|Omicron|EPI_ISL_11583342|	CAAT.....................GATTTCATACAAAC.........................GTAGATTCTTATTATTCATTGTTAA......................GGCT
	SARS-CoV-2|Omicron|EPI_ISL_11580532|	CAAT.....................GATTTCATACAAAC.........................GTAGATTCTTATTATTCATTGTTAA......................GGCT
	SARS-CoV-2|Iota|EPI_ISL_2958983|	CAAT.....................GATTTCATACAAAC.........................GTAGATTCTTATTATTCATTGTTAA......................GGCT
	SARS-CoV-2|-|EPI_ISL_8422346|	CAAT.....................GATTTCATACAAAC.........................GTAGATTCTTATTATTCATTGTTAA......................GGCT
Feline SARS-CoV-2	SARS-CoV-2|Alpha|EPI_ISL_4875726|	CAAT.....................GATTTCATACAAAC.........................GTAGATTCTTATTATTCATTGTTAA......................GGCT
	SARS-CoV-2|Alpha|EPI_ISL_6204713|	CAAT.....................GATTTCATACAAAC.........................GTAGATTCTTATTATTCATTGTTAA......................GGCT
	SARS-CoV-2|Delta|EPI_ISL_11583333|	CAAT.....................GATTTCATACAAAC.........................GTAGATTCTTATTATTCATTGTTAA......................GGCT
	SARS-CoV-2|Delta|EPI_ISL_11348757|	CAAT.....................GATTTCATACAAAC.........................GTAGATTCTTATTATTCATTGTTAA......................GGCT
	SARS-CoV-2|Delta|EPI_ISL_5320246|	CAAT.....................GATTTCATACAAAC.........................GTAGATTCTTATTATTCATTGTTAA......................GGCT
	SARS-CoV-2|Gamma|EPI_ISL_4565991|	CAAT.....................GATTTCATACAAAC.........................GTAGATTCTTATTATTCATTGTTAA......................GGCT
	SARS-CoV-2|Omicron|EPI_ISL_11610924|	CAAT.....................GATTTCATACAAAC.........................GTAGATTCTTATTATTCATTGTTAA......................GGCT
	SARS-CoV-2|Omicron|EPI_ISL_13647600|	CAAT.....................GATTTCATACAAAC.........................GTAGATTCTTATTATTCATTGTTAA......................GGCT
	SARS-CoV-2|Iota|EPI_ISL_2930559|	CAAT.....................GATTTCATACAAAC.........................GTAGATTCTTATTATTCATTGTTAA......................GGCT
	SARS-CoV-2|Epsilon|EPI_ISL_2930561|	CAAT.....................GATTTCATACAAAC.........................GTAGATTCTTATTATTCATTGTTAA......................GGCT
	SARS-CoV-2|Lambda|EPI_ISL_2791488|	CAAT.....................GATTTCATACAAAC.........................GTAGATTCTTATTATTCATTGTTAA......................GGCT
	SARS-CoV-2|-|EPI_ISL_437349|	CAAT.....................GATTTCATACAAAC.........................GTAGATTCTTATTATTCATTGTTAA......................GGCT
VOC variant	SARS-CoV-2|Alpha|EPI_ISL_3759431|	CAAT.....................GATTTCATACAAAC.........................GTAGATTCTTATTATTCATTGTTAA......................GGCT
	SARS-CoV-2|Beta|EPI_ISL_3832051|	CAAT.....................GATTTCATACAAAC.........................GTAGATTCTTATTATTCATTGTTAA......................GGCT
	SARS-CoV-2|Delta|EPI_ISL_4227161|	CAAT.....................GATTTCATACAAAC.........................GTAGATTCTTATTATTCATTGTTAA......................GGCT
	SARS-CoV-2|Gamma|EPI_ISL_4093614|	CAAT.....................GATTTCATACAAAC.........................GTAGATTCTTATTATTCATTGTTAA......................GGCT
	SARS-CoV-2|Omicron|EPI_ISL_11725714|	CAAT.....................GATTTCATACAAAC.........................GTAGATTCTTATTATTCATTGTTAA......................GGCT
Omicron subvariant	SARS-CoV-2|Omicron(BA2.12.1)|EPI_ISL_13521490|	CAAT.....................GATTTCATACAAAC.........................GTAGATTCTTATTATTCATTGTTAA......................GGCT
	SARS-CoV-2|Omicron(BA2.75)|EPI_ISL_14153192|	CAAT.....................GATTTCATACAAAC.........................GTAGATTCTTATTATTCATTGTTAA......................GGCT
	SARS-CoV-2|Omicron(BA4)|EPI_ISL_13409486|	CAAT.....................GATTTCATACAAAC.........................GTAGATTCTTATTATTCATTGTTAA......................GGCT
	SARS-CoV-2|Omicron(BA4.1)|EPI_ISL_13258801|	CAAT.....................GATTTCATACAAAC.........................GTAGATTCTTATTATTCATTGTTAA......................GGCT
	SARS-CoV-2|Omicron(BA4.1.1)|EPI_ISL_13700301|	CAAT.....................GATTTCATACAAAC.........................GTAGATTCTTATTATTCATTGTTAA......................GGCT
	SARS-CoV-2|Omicron(BA4.1.2)|EPI_ISL_13326783|	CAAT.....................GATTTCATACAAAC.........................GTAGATTCTTATTATTCATTGTTAA......................GGCT
	SARS-CoV-2|Omicron(BA5)|EPI_ISL_13537579|	CAAT.....................GATTTCATACAAAC.........................GTAGATTCTTATTATTCATTGTTAA......................GGCT
	SARS-CoV-2|Omicron(BA5.1)|EPI_ISL_13699954|	CAAT.....................GATTTCATACAAAC.........................GTAGATTCTTATTATTCATTGTTAA......................GGCT
	SARS-CoV-2|Omicron(BA5.2)|EPI_ISL_13360687|	CAAT.....................GATTTCATACAAAC.........................GTAGATTCTTATTATTCATTGTTAA......................GGCT
	SARS-CoV-2|Omicron(BA5.2.1)|EPI_ISL_13700716|	CAAT.....................GATTTCATACAAAC.........................GTAGATTCTTATTATTCATTGTTAA......................GGCT
	SARS-CoV-2|Omicron(BA5.3)|EPI_ISL_13067661|	CAAT.....................GATTTCATACAAAC.........................GTAGATTCTTATTATTCATTGTTAA......................GGCT
	SARS-CoV-2|Omicron(BA5.3.1)|EPI_ISL_13429728|	CAAT.....................GATTTCATACAAAC.........................GTAGATTCTTATTATTCATTGTTAA......................GGCT
	SARS-CoV-2|Omicron(BA5.3.2)|EPI_ISL_13474406|	CAAT.....................GATTTCATACAAAC.........................GTAGATTCTTATTATTCATTGTTAA......................GGCT
	SARS-CoV-2|Omicron(BA5.5)|EPI_ISL_13621811|	CAAT.....................GATTTCATACAAAC.........................GTAGATTCTTATTATTCATTGTTAA......................GGCT
VOI variant	SARS-CoV-2|Iota|EPI_ISL_2828312|	CAAT.....................GATTTCATACAAAC.........................GTAGATTCTTATTATTCATTGTTAA......................GGCT
	SARS-CoV-2|Eta| EPI_ISL_3908076|	CAAT.....................GATTTCATACAAAC.........................GTAGATTCTTATTATTCATTGTTAA......................GGCT
	SARS-CoV-2|Lambda|EPI_ISL_3215204|	CAAT.....................GATTTCATACAAAC.........................GTAGATTCTTATTATTCATTGTTAA......................GGCT
	SARS-CoV-2|Mu|EPI_ISL_3573839|	CAAT.....................GATTTCATACAAAC.........................GTAGATTCTTATTATTCATTGTTAA......................GGCT
	SARS-CoV-2|Kappa|EPI_ISL_2645187|	CAAT.....................GATTTCATACAAAC.........................GTAGATTCTTATTATTCATTGTTAA......................GGCT
	SARS-CoV-2|Theta|EPI_ISL_3026635|	CAAT.....................GATTTCATACAAAC.........................GTAGATTCTTATTATTCATTGTTAA......................GGCT
	SARS-CoV-2|Epsilon|EPI_ISL_4029296|	CAAT.....................GATTTCATACAAAC.........................GTAGATTCTTATTATTCATTGTTAA......................GGCT
	SARS-CoV-2|Zeta|EPI_ISL_13747907|	CAAT.....................GATTTCATACAAAC.........................GTAGATTCTTATTATTCATTGTTAA......................GGCT

Supplementary Figure 1

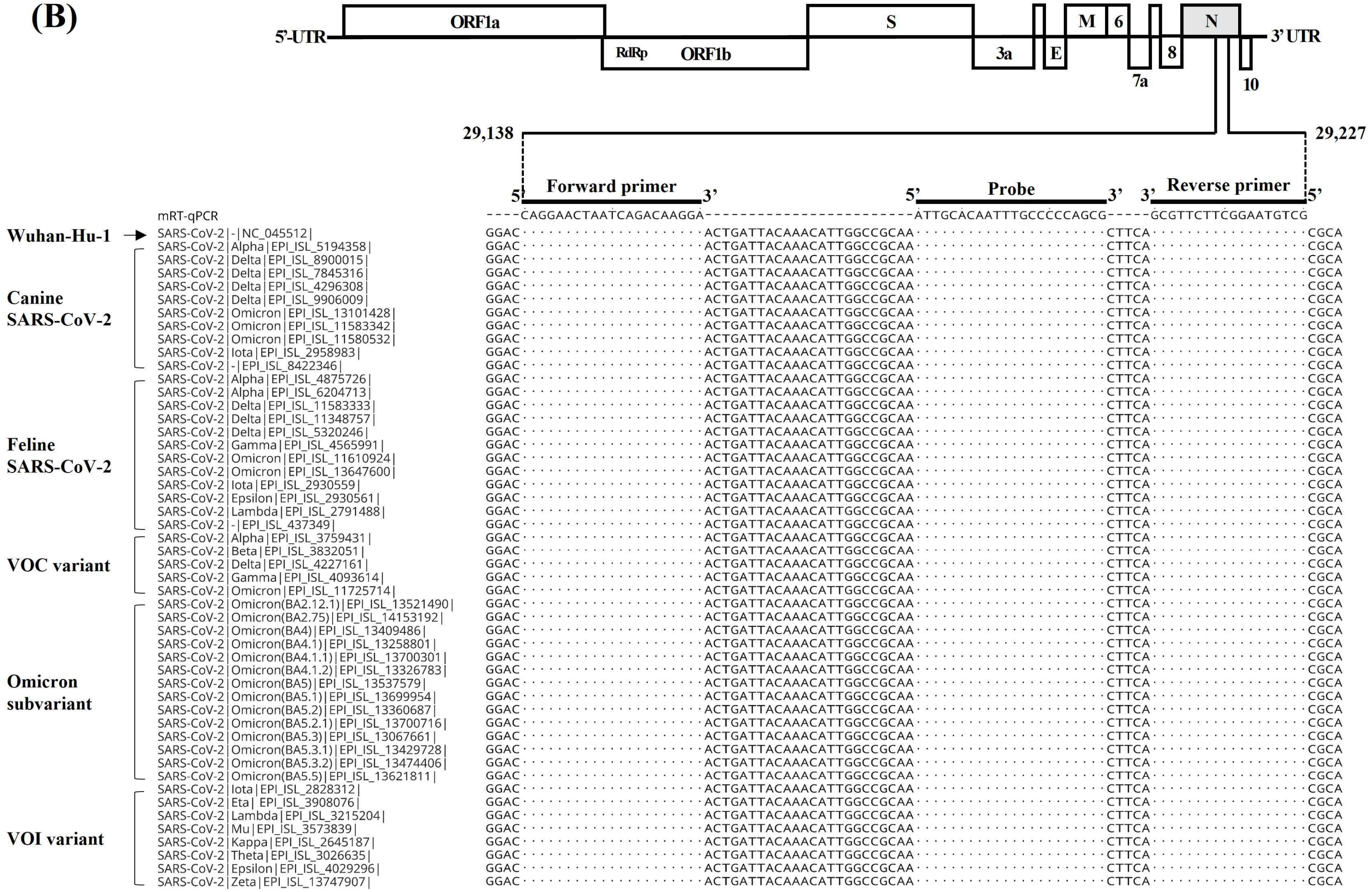

Figure S1. The alignment of newly designed *RdRp* and *N* gene primers and probe sequences with SARS-CoV-2 reference strain (Wuhan-Hu-1), canine SARS-CoV-2 sequences, feline SARS-CoV-2 sequences, human VOC, omicron subvariants and VOI variants sequences. Dot indicates the same base.

Supplementary Figure 2.

(A)

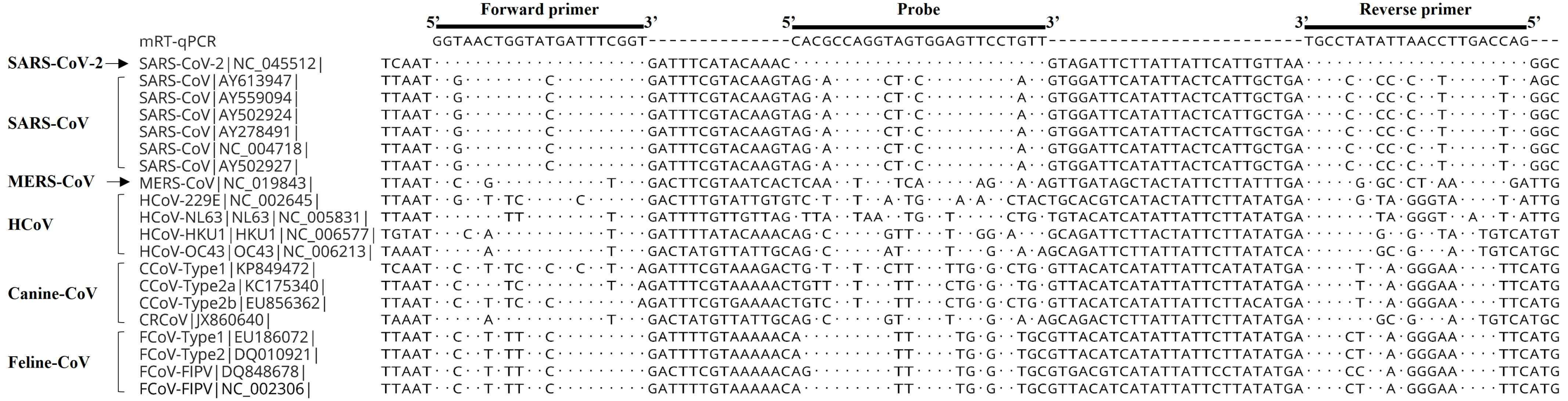

(B)

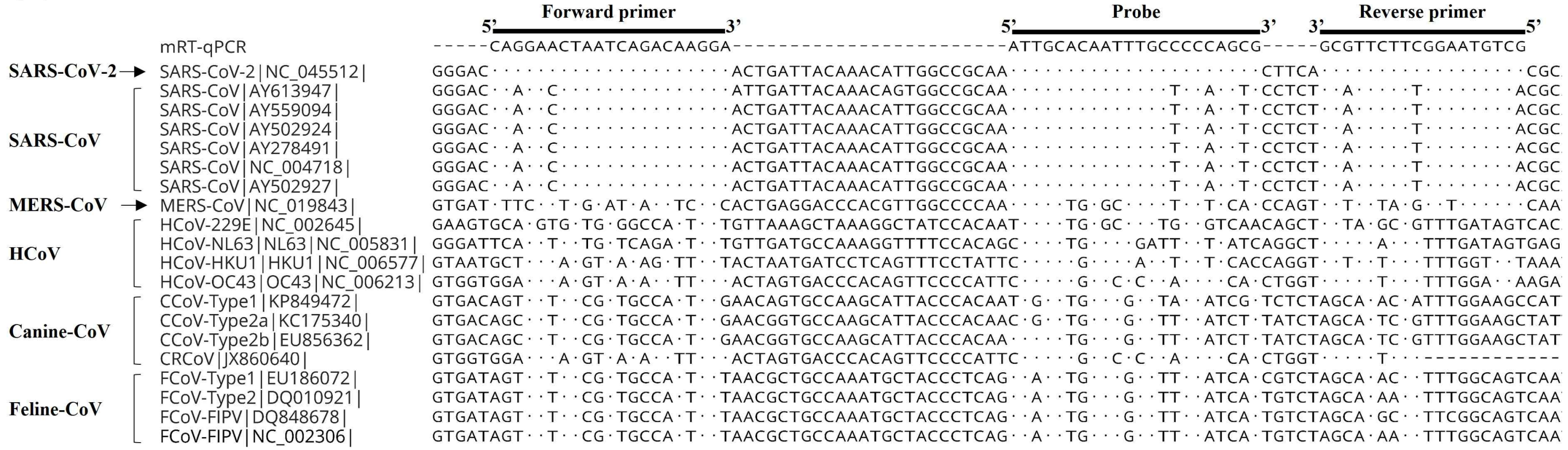

Figure S2. The alignment of the newly designed *RdRp* and *N* gene primers and probe sequences against SARS-CoV-2 reference strain (NC_045512), SARS-CoVs, MERS-CoV, HCoVs, Canine-CoVs and Feline-CoVs sequences. Dot indicates the same base. Bar indicates the deletion base.
