# Supplementary material for: Tailored Multiplex Real-Time RT-PCR with Species-Specific Internal Positive Controls for Detecting SARS-CoV-2 in Canine and Feline Clinical Samples
Source: Animals (Basel). 2023 Feb 9;13(4):602. doi: 10.3390/ani13040602 (PMC9951688; doi:10.3390/ani13040602)
Supplement: Supplementary file 1 [file animals-13-00602-s001.zip › Supplementary Table S1.docx]

Supplementary Table S1. List of abbreviations

| Abbreviation | Meaning |
| --- | --- |
| ADIC | Animal Disease Intervention Center |
| APQA | Animal and Plant Quarantine Agency |
| *B. bronchiseptica* | *Bordetella bronchiseptica* |
| BHQ | Black Hole Quencher |
| CAdV-2 | Canine adenovirus 2 |
| CAVS | Commercially available vaccine strain |
| CCoV | Canine coronavirus |
| CDV | Canine distemper virus |
| CI | Confidence interval |
| CIV | Canine influenza virus |
| COVID-19 | Coronavirus disease-2019 |
| CPIV | Canine parainfluenza virus |
| CPV | Canine parvovirus |
| Ct | Cycle threshold |
| CV | Coefficient of variation |
| Cy5 | Cyanine 5 |
| *E* | Envelope |
| EIPC | Endogenous internal positive controls |
| FAM | 6-carboxyfluorescein |
| FCV | Feline calicivirus |
| FeLV | Feline leukemia virus |
| FHV | Feline herpesvirus |
| *GAPDH* | Glyceraldehyde 3-phosphate dehydrogenase |
| GISAID | Global Initiative on Sharing All Influenza Data |
| HEX | 6-carboxy-2′,4,4′,5′,7,7′-hexachlorofluorescein |
| LOD | Limit of detection |
| Mean | Mean value |
| MIQE | Minimum Information for Publication of Quantitative Real-Time PCR Experiments |
| mRT-qPCR | Multiplex real-time quantitative polymerase chain reaction |
| *N* | Nucleocapsid |
| NCBI | National Center for Biotechnology Information |
| NCCP | National Culture Collection for Pathogens |
| *ORF1ab* | Open reading frame 1ab |
| *RdRp* | RNA-dependent RNA polymerase |
| RT-qPCR | Reverse transcription real-time quantitative polymerase chain reaction |
| RVSL | Regional veterinary service laboratory |
| *S* | Spike |
| SARS-CoV-2 | Severe acute respiratory syndrome coronavirus 2 |
| SD | Standard deviation |
| Tm | Melting temperature |
| VOC | Variants of concern |
| VOI | Variants of interest |
| VUM | Variants under monitoring |
| WHO | World Health Organization |
